# Bacterial metabolosomes: new insights into their structure and bioengineering

**DOI:** 10.1111/1751-7915.13740

**Published:** 2021-01-06

**Authors:** Lu‐Ning Liu

**Affiliations:** ^1^ Institute of Systems, Molecular and Integrative Biology University of Liverpool Liverpool L69 7ZB UK; ^2^ College of Marine Life Sciences Frontiers Science Center for Deep Ocean Multispheres and Earth System Ocean University of China Qingdao 266003 China

## Abstract

Bacterial metabolosomes have been discovered for over 25 years. They play essential roles in bacterial metabolism and pathogenesis. In this crystal ball paper, I will discuss the recent advances in the fundamental understanding and synthetic engineering of bacterial metabolosomes.

## Graphical abstract

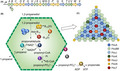

Self‐assembly and compartmentalization have been noticed as the ubiquitous phenomena in biology, physics and chemistry. Within cells, compartmentalization provides the structural basis for confining metabolic reactions in space and time. Compared with eukaryotic cells, prokaryotic cells have been thought to be simpler and less structurally defined for decades. It is now crystal clear that bacteria have complex, highly ordered subcellular structures and compartmentalized machines, such as outer membrane vesicles, intracellular thylakoids, chromatophores, membrane‐less organelles, as well as a range of regulatory systems that are involved in circadian clocks, motility, chemotaxis and secretion, etc. (Cannon *et al*., 2001; Mullineaux and Liu, 2020; Zhao *et al*., 2020).

A typical example of organelles spatially segregated in prokaryotic cells is the bacterial microcompartment (BMC), the special metabolically functional machinery that is widespread among bacterial phyla (Axen *et al*., 2014; Ravcheev *et al*., 2019). BMCs are formed entirely by proteins through ingenious self‐assembly of several thousand protein polypeptides of 10–20 different types. The organelles encapsulate a collection of cargo enzymes that catalyse sequential metabolic reactions within a polyhedral protein shell that structurally resembles virus capsids. The shell is typically around 100–200 nm in size and is built by various proteins in the forms of hexamers and trimers that form the shell facets and pentamers that cap the shell vertices (Kerfeld *et al*., 2018). The shell proteins are generally perforated by a central pore, and our recent computational simulations study has indicated their selective permeability that allows passage of substrates and products in and out of the organelle (Faulkner *et al*., 2020). Therefore, in addition to confining the interior organization and packing, the protein shell also acts as a physical barrier, enabling BMCs to modulate metabolic reactions that produce intermediates which are toxic or poorly retained by the cell envelope (Yeates *et al*., 2010; Bobik *et al*., 2015). The intriguing, natural designs of BMCs lay the foundation for their roles in CO_2_ fixation, pathogenesis and microbial ecology.

According to their distinct functions, BMCs can be categorized into anabolic BMCs (carboxysomes) and catabolic BMCs (metabolosomes). Carboxysomes are the most intensively studied group of BMCs that function as the key CO_2_‐fixing organelles in all cyanobacteria and many chemoautotrophs. Carboxysomes encapsulate carbonic anhydrase and the primary carboxylating enzymes, ribulose‐1,5‐bisphosphate carboxylase oxygenase (Rubisco) within the compartment. Depending on the types of encased Rubisco, carboxysomes can be divided into α‐carboxysomes and β‐carboxysomes. The shell facilitates accumulation of HCO_3_^‐^ that will be dehydrated by carbonic anhydrase to CO_2_ and prevents CO_2_ leakage into the cytosol. This allows the generation of a CO_2_‐rich microenvironment within the carboxysome to facilitate Rubisco carboxylation. Our recent studies have provided new insights into the assembly, structure, permeability, physiological regulation and bioengineering of carboxysomes (Sun *et al*., 2016; Faulkner *et al*., 2017; Fang *et al*., 2018; Huang *et al*., 2019; Sun *et al*., 2019; Faulkner *et al*., 2020; Huang *et al*., 2020; Li *et al*., 2020).

The term ‘metabolosome’ was initially used to refer to an RNA organizing structure that mediates the metabolism of catalytic RNA (Gibson and Lamond, 1990). Subsequently, catabolic BMCs that were found in various bacteria and archaea including human gut microbes were termed as metabolosomes (Brinsmade *et al*., 2005) to implicate their roles in the degradation of diverse carbon sources, such as but not limited to propanediol, ethanolamine, fucose and rhamnose. They were also termed as entersomes previously (Cannon *et al*., 2001). Among the bacterial metabolosomes, Pdu (1,2‐propanediol utilization) metabolosomes and Eut (ethanolamine utilization) metabolosomes in the pathogenic bacteria *Salmonella enterica* and *Escherichia coli* are the best‐studied metabolosomes. As a common feature, metabolosomes encase a core catabolic enzyme to generate acetaldehyde or propionaldehyde intermediates which are then converted to alcohol and acid derivatives (Juodeikis *et al*., 2020).

We now have an increasing understanding of the significance of metabolosomes in providing the metabolic advantage to the growth of bacteria in specific niches. *S. enterica* is a dangerous pathogen that causes food poisoning and massive gut inflammation. To overcome the nutrient limitation within the highly competitive gut environment caused by the intestinal microbiota and outcompete other organisms in the gut, *S. enterica* has evolved specific metabolic features to consume compounds that are not used by the commensal bacteria. For example, 1,2‐propanediol (1,2‐PD) is a major metabolite produced by the gut microbiota through fermentation of fucose or rhamnose and can serve as a growth substrate for *S. enterica* under both aerobic and anaerobic conditions. Therefore, degradation of 1,2‐PD by Pdu metabolosomes in *S. enterica* provides a fitness advantage that promotes the growth of *S. enterica* in the inflamed gut, which is essential for enteric pathogenesis. In addition, degradation of phosphatidylethanolamine derived from ingested food and turnover of the intestinal epithelium and residential microbiota allows ethanolamine to be particularly prevalent in the gastrointestinal tract (Kaval and Garsin, 2018). Ethanolamine is an important source of carbon and nitrogen for bacteria. The capability in ethanolamine catabolism gives a competitive advantage to *S. enterica* (Thiennimitr *et al*., 2011) and enterohemorrhagic *E. coli* (Bertin *et al*., 2011) in the inflamed intestine.

The naturally designed architecture of metabolosomes is vital for their metabolic activities. In *S. enterica*, the Pdu metabolosome is constructed by 22 types of proteins that are encoded by a single *pdu* operon (Fig. 1). The core enzymes include diol dehydratase (PduCDE), phosphotransacylase (PduL), aldehyde dehydrogenase (PduP), alcohol dehydrogenase (PduQ) and propionate kinase (PduW). PduCDE catalyses the conversion of 1,2‐PD to propionaldehyde, which is then converted to propionyl coenzyme A (propionyl‐CoA) or 1‐propanol by PduP or PduQ, respectively. PduL catalyses the conversion of propionyl‐CoA to propionyl‐phosphate, which is then converted into propionate by PduW to generate ATP. There are also other enzymes involved in the 1,2‐PD metabolism, such as cobalamin reductase (PduS), adenosyltransferase (PduO), diol dehydratase reactivase (PduGH) and L‐threonine kinase (PduX), for the reactivation of diol dehydratase and vitamin B_12_ recycling. The shell of the Pdu metabolosome contains nine proteins: PduA, B, B', M, N, J, K, T and U. The shell confines propionaldehyde to protect the bacterial cell from toxic sequelae. A regulatory protein PocR positively regulates the expression of the *pdu* operon.

**Fig. 1:**
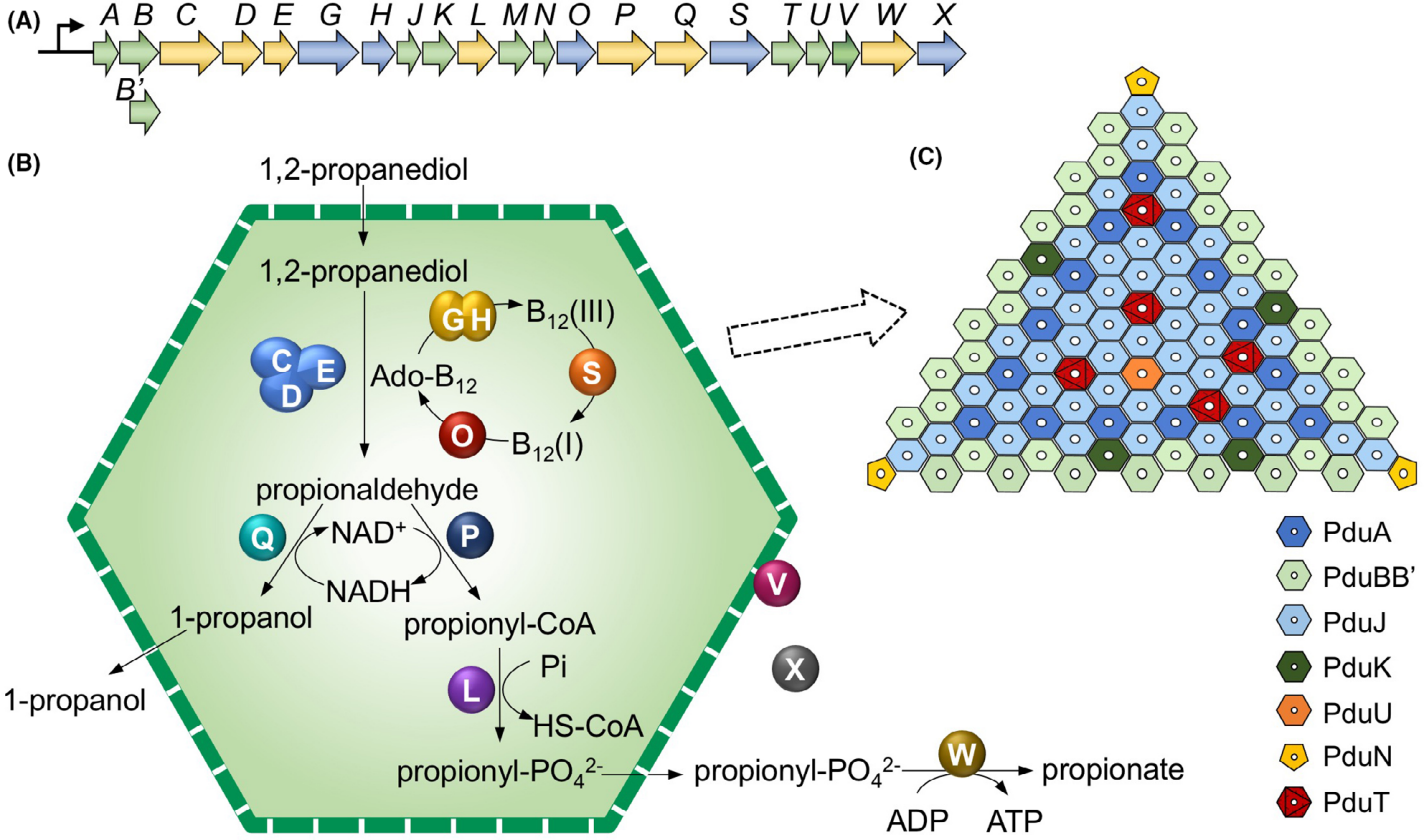
Gene organization and architectural models of the Pdu metabolosome and shell (Yang et al., 2020). A. diagram of the *pdu* operon that encodes proteins of Pdu metabolosomes. B. schematic model for the protein organization and metabolic pathways of entire Pdu metabolosome. C. structural model of one shell facet.

Eut metabolosomes share similar structural principles with Pdu metabolosomes. Within the *S. enterica* Eut metabolosome, adenosyl‐B_12_ (Ado‐B_12_)‐dependent ethanolamine ammonia lyase (EutBC) catalyses the first step of ethanolamine utilization to produce ammonia (a nitrogen source) and acetaldehyde that is converted into acetyl coenzyme A (acetyl‐CoA) by aldehyde dehydrogenase (EutE) or into ethanol by alcohol dehydrogenase (EutG). Phosphotransacetylase (EutD) catalyses the conversion of acetyl‐CoA to acetyl‐phosphate that leaves the metabolosome and subsequently is converted to acetate. This pathway leads to the generation of ATP under fermentative conditions, while under respiratory conditions the resulting acetyl‐CoA promotes cell growth via the TCA (tricarboxylic acid) cycle. The degradation of ethanolamine also requires two Ado‐B_12_ recycling enzymes EutA and EutT. There are also two acetate kinases EutP and EutQ that is required for anaerobic respiration of ethanolamine with tetrathionate, and an ethanolamine permease EutH that mediates transport of protonated ethanolamine. Five shell proteins (EutS, EutM, EutN, EutL and EutK) are involved in the Eut metabolosome, along with a putative chaperone EutJ. A positive regulatory protein EutR regulates the transcription of the *eut* operon in the presence of both ethanolamine and Ado‐B_12_.

One long‐standing question in the studies of bacterial metabolosomes is their actual stoichiometric composition and protein arrangement. Our recent work has reported the accurate stoichiometry of shell proteins and internal enzymes of the natural Pdu metabolosomes from *S. enterica*, using QconCAT‐driven quantitative mass spectrometry (Yang *et al*., 2020). This has led us to develop a structural model of the Pdu metabolosome (Fig. 1), which provides essential insight into the architectural principles of metabolosomes and synthetic engineering of functional metabolosome structures. In addition, by genetically deleting one major shell protein PduA from the native Pdu metabolosomes, we characterized the changes in shell protein organization and cargo enzyme content of Pdu metabolosome variants. The finding provided direct evidence regarding the structural remodelling of metabolosomes and specific protein–protein interactions that define the metabolosome architecture. Consistently, our study on the protein stoichiometry of anabolic carboxysomes also indicated the structural variation of carboxysomes in response to environmental changes during cell growth (Sun *et al*., 2019). Thus, the structural variability appears to be a common feature of bacterial microcompartment assembly.

What is the overall three‐dimensional architecture of bacterial metabolosomes? Numerous studies have reported the structures of intact carboxysomes from different origins (Iancu *et al*., 2007; Ting *et al*., 2007; Iancu *et al*., 2010; Hantke *et al*., 2014; Faulkner *et al*., 2017; Dai *et al*., 2018). In contrast, the detailed structures of native metabolosomes remain murky, probably due to their highly flexible and heterogeneous structures. Nevertheless, recent crystallographic and cryo‐electron tomographic structures of recombinant empty metabolosome shells, which were produced in *E. coli* by expressing a selected set of shell components, have provided insights into metabolosome shell assembly and general structural principles (Sutter *et al*., 2017; Greber *et al*., 2019; Kalnins *et al*., 2020). One recombinant shell was derived from the metabolosome of the myxobacterium *Haliangium ochraceum*, which consists of one hexamer, one pentamer and three trimeric shell proteins (Sutter *et al*., 2017). The formed synthetic shell possesses an icosahedral shape, in which hexamer and trimers form the flat facets while pentamers occupy the vertices. Structural analysis of the synthetic shell clarified the sidedness of shell proteins in the shell structure⏤the concave sides of shell proteins face outward. Further work revealed the structural variations of the *H. ochraceum* recombinant shell, implicating that the interactions mediating shell formation have a certain level of plasticity (Greber *et al*., 2019). This plasticity may play roles in metabolosome shell assembly and permeability in native hosts. The overall protein organization of the shell structure is consistent with the cryo‐EM structure of recombinant GRM2 metabolosome shell (containing CmcABC′D) from *Klebsiella pneumoniae*, which are involved in choline utilization (Kalnins *et al*., 2020). It is worthy to note that these synthetic shells exhibited the much smaller and more regular structures than wild‐type metabolosomes.

How do the protein components assemble to form BMCs? It has been characterized that *de novo* assembly of β‐carboxysomes exploits the ‘inside out’ model (Cameron *et al*., 2013): Rubisco and CcmM forming the core first, followed by encapsulation of shell proteins. This model has been verified by our recent study on Rubisco accumulation factor 1 (Raf1)‐mediated Rubisco biogenesis and β‐carboxysome assembly (Huang *et al*., 2020) and could explain the higher Rubisco packing density in β‐carboxysome (Faulkner *et al*., 2017). For the α‐carboxysome, we have recently demonstrated that intact α‐carboxysome shells can be reconstructed in *E. coli* and external enzymes can be encapsulated within the shell, indicating that the α‐carboxysome shell and enzymes likely assemble concomitantly. This was substantiated by previous EM observations of partially assembled α‐carboxysome shells with attached core proteins *in vivo* (Iancu *et al*., 2010; Dai *et al*., 2018). Using computational simulations, both concomitant and ‘inside out’ assembly modes have been examined (Perlmutter *et al*., 2016; Rotskoff and Geissler, 2018). The different assembly manners are probably dependent on the strength of cargo–cargo interactions. The assembly pathway of metabolosomes and whether metabolosomes assemble following the concomitant mode like α‐carboxysome or via the ‘inside out’ mode like β‐carboxysomes have yet to be experimentally validated.

Advance knowledge of BMC assembly and function has allowed us to start the exploration of reprogramming BMC structures for biotechnological and biomedical applications in metabolic improvement, molecule delivery and therapeutics. It was shown that expressing the entire Pdu operon including 21 genes from the anaerobic gram‐negative bacterium *Citrobacter freundii* in the *E. coli* host cells could produce functional metabolosomes that can carry out propanediol utilization (Parsons *et al*., 2008). It was further demonstrated that Pdu metabolosome shells can be formed without cargo enzymes, although possessing a smaller size than the wild‐type metabolosome (Parsons *et al*., 2010). These studies, together with the identification of encapsulation peptides and development of new encapsulation strategies (Hagen *et al*., 2018; Lee *et al*., 2018a), provided the foundation for repurposing metabolosome shells to encase external enzymes, such as pyruvate decarboxylase and alcohol dehydrogenase for ethanol production (Lawrence *et al*., 2014), glycerol dehydrogenase, dihydroxyacetone kinase, methylglyoxal synthase and 1,2‐propanediol oxidoreductase for 1,2‐PD production (Lee *et al*., 2016), as well as polyphosphate kinase for accumulation of polyphosphate within the metabolosome shell (Liang *et al*., 2017). Very recently, we have shown that empty α‐carboxysome shells (about 100 nm in diameter) can be reconstructed by expressing the full set of shell proteins and can recruit mature O_2_‐sensitive [FeFe]‐hydrogenase and cofactors into the shell to create a novel hydrogen‐producing as a novel nanoscale bioreactor, taking advantage of the O_2_‐free (or O_2_‐less) microenvironment created within the α‐carboxysome shell (Li *et al*., 2020). The dimension and shape of the recombinant α‐carboxysome shell are comparable to those of native carboxysomes, suggesting the improved enzyme loading and more close‐to‐native microenvironment compared with the shells with the reduced size.

Expressing individual shell proteins can also lead to formation of diverse higher‐order assemblies, e.g. two‐dimensional flat sheets, nanotubes and filament structures. We have applied high‐speed atomic force microscopy to visualize the dynamic assembly process of shell hexamers from *H. ochraceum* and demonstrated that shell proteins in the shell facets have the same sidedness (Sutter *et al*., 2016). We have further proved that the assembly of shell proteins could be affected when the pH and salt conditions change, indicative of the possible ways to modulate shell protein assembly and disassembly *in vitro* (Faulkner *et al*., 2019). Interestingly, the synthetic nanotube structure formed by PduA was shown to have the capacity of accommodating pyruvate decarboxylase and alcohol dehydrogenase as synthetic scaffolds to facilitate ethanol production (Lee *et al*., 2018b).

Despite many exciting research advances, there are still open questions in the fundamental understanding and bioengineering of bacterial metabolosomes, for example, what the assembly pathways of distinct metabolosomes are, how metabolosomes are segregated and maintained in bacterial cells, how cargos are organized and interact with other cofactors within the shell, and how to control self‐assembly and shell encapsulation. Additionally, it is often that two or more types of metabolosomes coexist within one bacterium. How are their expression, assembly and function precisely coordinated in the same cell? On the other hand, the high similarity of shell protein structures and the shared building principles of BMCs offer the potential for rational design and engineering of chimeric BMC structures, with the capacity of fine‐tuning shell permeability and catalytic efficiency. Further efforts are also needed to gain an in‐depth understanding of the roles and controls of metabolosomes in pathogenesis and human health.

## Conflict of interest

The author declares no conflict of interest.
